# Discriminability of multiple cutaneous and proprioceptive hand percepts evoked by intraneural stimulation with Utah slanted electrode arrays in human amputees

**DOI:** 10.1186/s12984-021-00808-4

**Published:** 2021-01-21

**Authors:** David M. Page, Jacob A. George, Suzanne M. Wendelken, Tyler S. Davis, David T. Kluger, Douglas T. Hutchinson, Gregory A. Clark

**Affiliations:** 1 Avanos Medical, Alpharetta, GA 30004 USA; 2 Division of Physical Medicine and Rehabilitation, University of Utah, Salt Lake City, UT 84112 USA; 3 Department of Anesthesiology, Maine Medical Center, Portland, ME 04102 USA; 4 Department of Neurosurgery, University of Utah, Salt Lake City, UT 84112 USA; 5 Blackrock Microsystems, Salt Lake City, UT 84108 USA; 6 Department of Orthopaedics, University of Utah, Salt Lake City, UT 84112 USA; 7 Department of Biomedical Engineering, University of Utah, Salt Lake City, UT 84112 USA

**Keywords:** Neuromodulation, Neural interface, Brain computer interface, Peripheral nerve stimulation, Utah slanted electrode array, Sensory feedback, Neuroprostheses, Neural prosthesis, Bionic arm, Amputee

## Abstract

**Background:**

Electrical stimulation of residual afferent nerve fibers can evoke sensations from a missing limb after amputation, and bionic arms endowed with artificial sensory feedback have been shown to confer functional and psychological benefits. Here we explore the extent to which artificial sensations can be discriminated based on location, quality, and intensity.

**Methods:**

We implanted Utah Slanted Electrode Arrays (USEAs) in the arm nerves of three transradial amputees and delivered electrical stimulation via different electrodes and frequencies to produce sensations on the missing hand with various locations, qualities, and intensities. Participants performed blind discrimination trials to discriminate among these artificial sensations.

**Results:**

Participants successfully discriminated cutaneous and proprioceptive sensations ranging in location, quality and intensity. Performance was significantly greater than chance for all discrimination tasks, including discrimination among up to ten different cutaneous location-intensity combinations (15/30 successes, *p* < 0.0001) and seven different proprioceptive location-intensity combinations (21/40 successes, *p* < 0.0001). Variations in the site of stimulation within the nerve, via electrode selection, enabled discrimination among up to five locations and qualities (35/35 successes, *p* < 0.0001). Variations in the stimulation frequency enabled discrimination among four different intensities at the same location (13/20 successes, *p* < 0.0005). One participant also discriminated among individual stimulation of two different USEA electrodes, simultaneous stimulation on both electrodes, and interleaved stimulation on both electrodes (20/24 successes, *p* < 0.0001).

**Conclusion:**

Electrode location, stimulation frequency, and stimulation pattern can be modulated to evoke functionally discriminable sensations with a range of locations, qualities, and intensities. This rich source of artificial sensory feedback may enhance functional performance and embodiment of bionic arms endowed with a sense of touch.

## Background

Most commercially-available upper-limb prostheses do not provide amputees with sensory feedback. Sensation from a prosthesis has been shown to be important for performance of functional tasks and for prosthesis embodiment [1–4], and amputees indicate interest in having sensory feedback from their prosthesis [5–10]. Peripheral-nerve interface approaches, such as Utah Slanted Electrode Arrays (USEAs) [3, 4, 11–15], cuff electrodes [16, 17], transverse intrafascicular multichannel electrodes [1, 18–22], flat interface nerve electrodes [23–26], and longitudinal intrafascicular electrodes [27–30] have demonstrated the ability to evoke sensory percepts at different locations, and of different qualities (e.g., submodalities) and intensities on the missing hand of amputees. These sensory percepts have been shown to be important for identifying objects of different shapes/sizes and compliances during closed-loop prosthesis control [1, 4, 11, 29, 31–33].

The discriminability (greater than chance) of the evoked sensory percepts is critical for closed-loop prosthesis control. Discriminability among sensory percepts at different locations and with different qualities has been reported for cuff electrodes [34], transverse intrafascicular multichannel electrodes [21, 31, 35], and flat interface nerve electrodes [36]. However, reports on the discriminability of USEA-evoked percepts have been limited to a small number of percepts [11], despite the fact that USEAs can evoke numerous sensory percepts [4, 13].

The USEA provides intrafascicular access to nerve fibers spanning the cross-section of a peripheral nerve via 100 penetrating microelectrodes. In contrast to other peripheral nerve interfaces, USEAs offer cross-sectional nerve access via many channels, enabling activation of numerous sensory percepts spanning the hand [4]. The selection of different USEA electrodes enables activation of different axons or subsets of axons with different projected field locations on the hand and with different sensory qualities. The intensity of each percept can be encoded based on the amplitude or frequency of stimulation [4]. Despite this understanding, prior publications using USEAs have not fully tested the extent to which human subjects can discriminate among multiple proprioceptive and cutaneous sensory percepts at different locations, and of different qualities and intensities, such as would be desirable during multi-sensor closed-loop prosthesis control.

The impact of increasing the resolution of sensory percepts on the functionality and naturalism of artificially evoked sensory feedback has not been documented empirically. However, discrimination tasks in intact human hands suggest that encoding of many locations, qualities and intensities, potentially via different receptor subtypes, is likely needed to recreate the full sensory experience of the human hand. For example, cutaneous location discrimination in the intact hand has been performed previously via a 2-point discrimination task, in which discriminability (greater than chance alone) was achievable for stimuli as close as 0.55 mm apart on the fingertips [37]. This high level of discriminability is likely attributable to intensity encoding via a population of afferents around the site of applied tactile pressure (receptor density is on the order of 1 per square millimeter on the palmar hand [38, 39]). Natural activation patterns in the human hand include activation of several different cutaneous mechanoreceptor subtypes innervating many different locations on the hand. The number of discriminable locations on an intact human hand has not been formally quantified, but, on the basis of these prior publications, is likely on the order of hundreds of sensory locations.

In microneurography studies, intact subjects have also discriminated among tactile percepts with the same location, but with different intensities. A roughly linear, nearly threefold increase in perceived intensity was noted both for normal cutaneous forces between 1–5 N and tangential forces between 1 and 3 N [40], with an informal indication that subjects are likely capable of discriminating up to ~ 10 different constant-force levels within these ranges. Constant-force intensities are generally accepted as being primarily encoded in the firing rates and activation patterns of Type-I slowly-adapting receptors (e.g., Merkel disk receptors) [41–44], although many receptor subtypes are generally activated during naturalistic touch of an intact hand. Type-I and Type-II rapidly-adapting cutaneous mechanoreceptors (i.e., Meissner and Pacinian corpuscles) are generally assumed to be the primary encoders of vibratory intensities via their population activation patterns and firing rates [41]. Human subjects have also been able to differentiate among at least 4 different amplitudes of vibratory tactile stimuli which were encoded with the amplitude (2.4–154 μm indentation) and frequency (10–200 Hz) of vibration [45].

Prior work has documented the discriminability of intensity for peripheral nerve stimulation using classic psychophysical methods (i.e., the just-noticeable difference) for cuff electrodes [16], transverse intrafascicular multichannel electrodes [21], flat interface nerve electrodes [24] and USEAs [46]. By extrapolation of just-noticeable differences across the presumed range of perceivable stimulus intensities, these results suggest that at least 15, and possibly up to 46, different intensities could be felt by modulating stimulation frequencies between 1 and 300 Hz. However, it is unclear whether these estimates can be extrapolated to hundreds of USEA-evoked percepts. For example, multi-channel intraneural stimulation with transverse intrafascicular multichannel electrodes has been shown to lower the amount of stimulation needed to evoke detectable sensory percepts [35], and this sensory facilitation may alter the just-noticeable differences and expand the range of stimulation current. Multi-channel intraneural stimulation with transverse intrafascicular multichannel electrodes yields a linear summation of sensation locations, suggesting minimal multi-electrode interactions and limited current summation [35]. However, current summation and non-linear responses have been documented for USEAs [47], and these are likely due to the higher electrode density and smaller inter-electrode distances that allow subthreshold currents from individual USEA electrodes to summate.

Here, we build on these prior studies by demonstrating discrimination among USEA-evoked percepts in blinded trials where subjects are presented with multiple possible stimulation conditions. These results highlight discrimination among four levels of intensity, eight locations, and ten location-intensity combinations for cutaneous percepts. We also highlight up to seven proprioceptive digit-position combinations, a unique finding given the relative scarcity of evoking proprioceptive percepts via other neural interfaces [16, 20, 23]. Furthermore, we demonstrate, for the first time, the ability to leverage the high electrode density of the USEA to evoke discriminable percepts by quality alone (i.e., distinct percept qualities with the same perceptive location) and to create linear or non-linear summations of percepts with interleaved or simultaneous multi-electrode stimulation. These results constitute an important step towards the development of sensorized bionic arms with a greater number of discriminable cutaneous and proprioceptive percepts.

## Methods

### Volunteers

Three transradial amputees participated in this study, referred to as S3, S4, and S5. Subject S3 was a 50-year-old male with a left-arm amputation that had occurred 21 years prior. Subject S4 was a 36-year-old male with bilateral amputations that had occurred 16 years prior. Subject S5 was a 43-year-old male with bilateral amputations that had occurred 24 years prior. Each subject underwent psychological and medical assessments prior to participating in the study. Subjects were provided with training materials prior to implantation of the electrodes to allow them to learn the concepts and methods of the experiments in advance and thus reduce post-implant training time. These included mirror-box or prosthesis-video training materials [48] as reported with previous subjects [13–15]. The subjects were monitored for medical risks both during and after the implant period, and subjects S4 and S5 were treated for implant-related infections that resolved without issue. The consenting process and experimental procedures were approved by the University of Utah Institutional Review Board, and the Department of the Navy Human Research Protection Program.

### Device

Two USEAs (Blackrock Microsystems, Salt Lake City, UT, USA) were implanted in each subject: one in the median nerve and one in the ulnar nerve. For each subject, the surgical implantation was completed in roughly one to two hours. The implant location for subject S3 was in the left forearm distal to the elbow, whereas the implants for subject S4 and S5 were placed midway along the left upper arm, proximal to the medial epicondyle and to many motor branch points, thereby providing access to muscle proprioceptive afferents from muscle spindles and Golgi tendon organs. USEAs consisted of 100 silicon microelectrodes (sputtered iridium oxide) spaced 400 μm apart in a 10 × 10 grid across a 4 × 4 mm square base. The electrodes varied from ~ 0.75 to 1.5 mm in length to allow cross-sectional access to fibers at different depths within the peripheral arm nerves [47]. The stimulation surface area for each electrode tip is estimated to be 1573 µm^2^ [49]. Separate looped platinum wires were also implanted as stimulation return leads and for use as recording reference and ground leads. These looped platinum wires were placed close to (within ~ 5 mm of) the USEAs at the time of implantation, and were generally sutured to the epineurium along with the USEA lead wires within a centimeter of each USEA [12]. Electrical connection to each USEA electrode was available via an external printed circuit board that was coupled percutaneously to USEAs via a bundle of gold lead wires. Connection of the external circuit board to stimulation and recording hardware was made via a ZIF-Clip-96 connector cable (Tucker-Davis Technologies Inc., Alachua, FL, USA) for S3 and S4, or a 96-channel Gator connector cable (Ripple LLC, Salt Lake City, UT, USA) for S5.

**Fig. 1:**
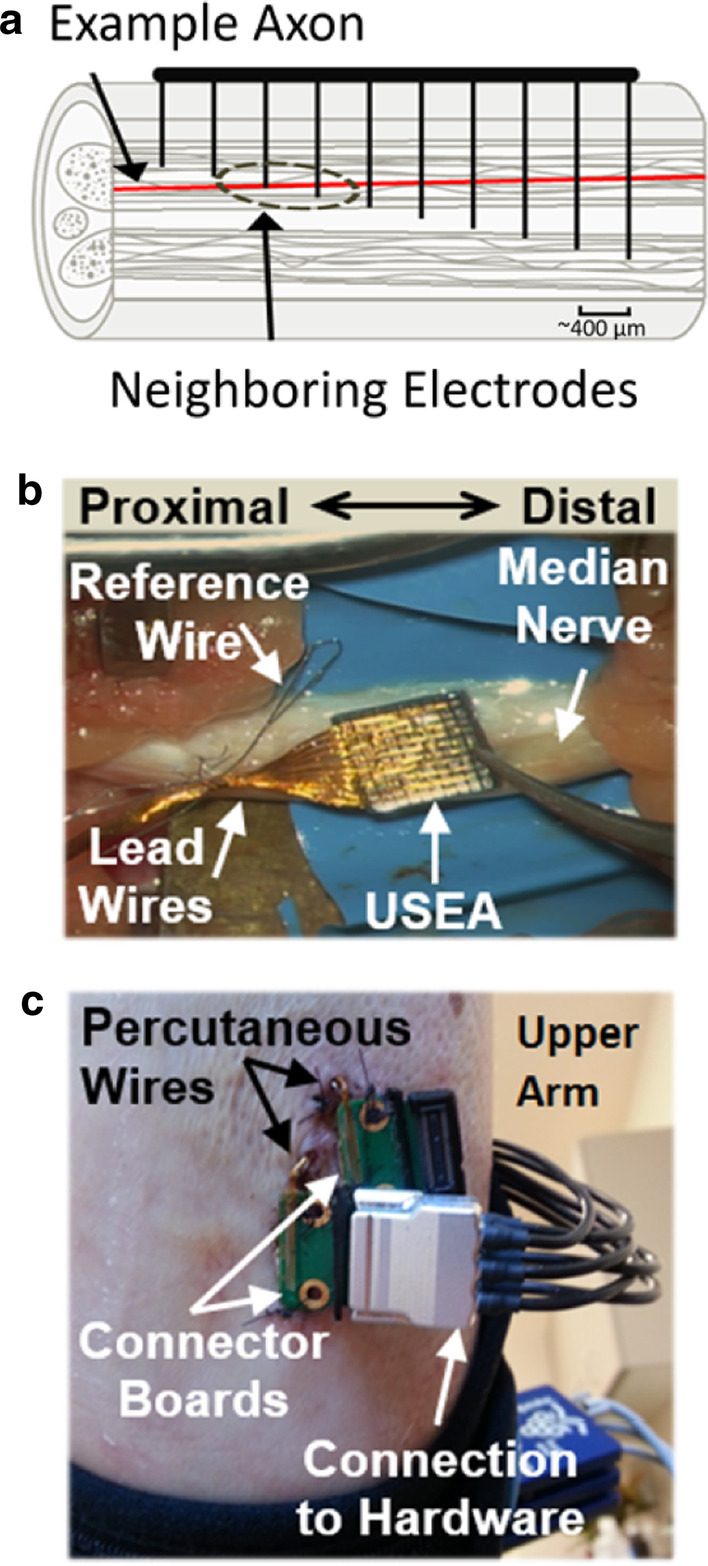
USEAs. **a** Absolute electrode distance versus cross-sectional projection distance. The 10 × 10 USEA provides cross-sectional coverage of peripheral nerves, increasing the possibility of activating different axons or subsets of axons with stimulation of each different electrode. Activation of different populations of axons is important for evoking sensory percepts with different locations or qualities. This diagram depicts a USEA implanted in a section of nerve, with an example axon that passes nearby two neighboring electrodes. Although the absolute distance between USEA electrodes is important for assessing stimulation selectivity limits, the cross-sectional distance between electrode tips more precisely indicates the likelihood that electrode tips are close to the same axon(s). For example, there is a ~ 409 μm absolute distance compared with a ~ 400 μm horizontal distance in and a ~ 83 μm vertical distance, not counting the exposure length of the electrode tip itself. USEA implant methods. **b** Photograph of a USEA in the median nerve of subject S4 taken shortly after pneumatic insertion. The bundle of gold lead wires as well as the separate ground and reference wires were later bundled to the nerve using a collagen nerve wrap. The USEAs were implanted with the long electrodes distally, to avoid damaging axons that may be recruited via stimulation of other USEA electrodes. **c** The USEA lead wires and ground and reference wires for each USEA (one in the median nerve; one in the ulnar nerve) remained attached to external connector boards via percutaneous incisions on either the lower or upper arm (subject S3 lower arm, subjects S4 and S5 upper arm). Stimulation hardware was attached to one or more of these external connectors during experimental sessions

The slanted nature of the USEAs enables cross-sectional nerve access to fibers at different depths, thereby increasing the possibility of activation of different axons or subsets of axons with each electrode [47]. An effort was made during the implant surgery to implant USEAs into the nerves so that the electrodes were positioned squarely perpendicular to the length of the nerve, which maximizes the cross-sectional nerve coverage of the USEA electrodes. The two-dimensional distance between two electrodes on the cross-sectional projection plane is likely the most influential factor on their ability to activate different axons or subsets of axons (Fig. 1a). The stimulation amplitude on a given electrode influences which axons near the tip of the electrode are activated, whereas the stimulation frequency influences their firing rate. The stimulation amplitude may also influence firing rate when modulated at peri-threshold levels, for example, when only a subset of stimulation pulses in a pulse train result in generation of an action potential [24], or when increasing stimulation intensity actives new fibers.

### Surgical and experimental procedures

Subjects were given prophylactic antibiotics the day before, the day of, and for several days following the implant surgery (100 mg minocycline, 7 days, twice per day). USEAs were implanted in each subject under general anesthesia, via similar methods to those described in past publications [12]. For subject S5, intramuscular electromyographic recording electrodes (Ripple Neuro LLC, Salt Lake City, UT, USA) were also placed in the muscles of the forearm for recording purposes, as described in [11, 12]. The intramuscular electromyographic recording electrodes were not used in the experiments presented herein. After exposure of each nerve implant site, the epineurium was dissected away, and USEAs were inserted into the nerve using a pneumatic insertion tool (Blackrock Microsystems, Salt Lake City, UT, USA) [50]. USEA lead wires and reference and ground wires were sutured to the epineurium, and a collagen wrap (AxoGen Inc., Alachua, FL, USA) was secured around the USEA, nerve, and reference and ground wires using vascular clips (Fig. 1b). For subject S5, the epineurium was sutured around the USEAs and reference and ground wires prior to placement of the collagen wrap. Dexamethasone (0.1 mg/kg) was delivered intravenously to the subjects during surgical closure as a potential means for decreasing the foreign body response [51, 52].

The site of percutaneous wire passage (Fig. 1c) was redressed roughly once per week using an antibiotic wound patch (Biopatch, Ethicon US LLC, Somerville, NJ, USA). The percutaneous connector boards were sutured to the skin in an attempt to stabilize the percutaneous wire passage, although this approach was abandoned for subjects S6 and S7 (not reported here). Subjects S4 and S5 both experienced infections at the USEA percutaneous wire passage site with subsequent full recoveries after USEA extraction and antibiotic treatment. Implants were removed after 4 weeks, 5 weeks, and 13 weeks, for S3, S4, and S5, respectively. The USEAs from subject S3 were removed along with the section of implanted neural tissue for histological analysis [53].

Experimental sessions were typically carried out 1–3 days per week, for 1–5 h each. Experimental sessions started 1–2 weeks after implant surgery and were performed throughout the remainder of implant durations (total implant durations of 4 weeks for S3, 5 weeks for S4, and 13 weeks for S5). In addition to the stimulation-evoked sensory percepts reported here, experimental sessions consisted of impedance testing, decoding of neuronal and myoelectric signals for prosthesis movement control, and closed-loop control of a prosthetic hand. Only the discrimination tasks for evoked sensory percepts are reported here.

### Microstimulation

Electrical stimulation was delivered using the IZ2-128 System (Tucker-Davis Technologies Inc., Alachua, FL, USA) for S3 and S4, or the Grapevine System (Ripple LLC, Salt Lake City, UT, USA) for S4 and S5. Stimulation was delivered as symmetric biphasic (cathodic-first) 200-µs square-wave pulses separated by a 100-µs interphase interval. Stimulation amplitudes and frequencies were kept below 120 µA and 500 Hz.

During discrimination trials for subjects S3 and S4, a single 200-ms train of stimulation was delivered at 200 Hz each time the subject or an experimenter pressed a button. For subject S5, three or four 500-ms trains of 100-Hz stimulation (unless noted otherwise, such as during intensity-encoding sessions) were delivered at a 50% duty cycle after the subject or the experimenters pressed a button. The 200-ms and 500-ms durations were selected as the minimal duration that the participants could still reliably perceive and distinguish the percepts; informally the participants felt their performance was not hindered by the duration of the stimulation trains, which is consistent with prior work showing that tactile mechanoreceptors are most active during onset of contact [43]. Shorter train durations in turn minimized sensory adaptation, as described in [26], across extended use (i.e., approximately one hour of repeated stimulation on a subset of electrodes for sensory discrimination tasks). Similarly, 100-Hz stimulation was used for subject S5 to minimize sensory adaptation. All discrimination trials were performed at an amplitude greater than the detection threshold that was comfortable and reliably perceivable (generally 5–10 µA above detection threshold). The 100-Hz and 200-Hz stimulation frequencies were selected because they had been used previously [13].

All stimulation parameters were approved by the University of Utah IRB and have been used with USEAs in multiple subjects before without any considerable neural damage [53]. A summary of stimulation parameters is shown in Table 1.

**Table 1:** Stimulation parameters

Stimulation parameter	Value
Electrode surface area	1573 µm^2^ [49]
Waveform shape	Symmetric, bi-phasic, cathodic-first, square-wave pulses
Duration of cathodic (stimulating) phase	200 µS
Duration of anodic (reversal) phase	200 µS
Inter-phase delay	100 µS
Current of cathodic (stimulating) phase	7–64 µA (unique and fixed for each electrode)
Current of anodic (reversal) phase	Equal magnitude as cathodic phase
Stimulation pulse frequency (during location/quality discrimination)	S3: 200 Hz
	S4: 200 Hz
	S5: 100 Hz
Train duration	S3: 200 ms
	S4: 200 ms
	S5: 500 ms
Number of trains	S3: 1
	S4: 1
	S5: 3–4
Duty cycle	S3: N/A
	S4: N/A
	S5: 50%

### Percept identification and mapping

We mapped the location, size, quality, intensity and detection threshold of USEA-evoked percepts over time, as detailed previously [4, 54]. Detection thresholds were established first. For each electrode individually, stimulation current was increased in 1–10-µA steps until a sensation was reliably detected (i.e., the blinded subject could correctly identify when stimulation was provided by the experimenter with 100% accuracy). The subjects then used custom software to indicate the location, size, quality, and intensity of the USEA-evoked sensory percept on the image of a hand. Subjects selected percept qualities from a list (e.g., pressure, vibration, tingle, etc.) or created their own descriptors as necessary. For cutaneous percepts, participants marked the location and size of the percept on an image of the hand; representations of percept locations and sizes were created based on the subjects’ software markings as well as their verbal descriptions as appropriate. For proprioceptive percepts, the subjects identified a joint that had moved or was moving due to the stimulation, and then selected their own angles to describe the degree of flexion felt on the joint (where 0° is fully extended and 180° is fully flexed).

These complete USEA percept maps (for ~ 200 electrodes) provided a basis for selection of the electrodes used in the discrimination trials reported here. The electrodes chosen for a given discrimination task were typically selected from complete USEA percept maps gathered within a week prior. Electrodes were chosen which provided distinct percepts with the desired location and/or quality. Prior to discrimination trials, the detection thresholds of the individual electrodes were verified, and then exceeded (by 5–10 µA) to ensure the percepts were reliably detected. Adaptation of sensory percepts, as described in [26], and electrode impedance were not used as a criterion for selection. The subjects were told to report any changes in percept location, size, quality or intensity of the percepts that may occur throughout the discrimination trials; no changes were reported by the subjects during the trials.

### Discrimination trials

Discrimination tasks were performed during different stimulation sessions. Discrimination tasks were unique to each subject; different subjects did not complete the same discrimination task due to limited time with the subjects and due to variations in the number and type of evoked percepts across participants and over time. A stimulation session typically included mapping the percept locations, qualities, and intensities associated with several USEA electrodes, and then down selecting to electrodes and stimulation frequencies that represented a subset of locations, qualities, intensities, or combinations for formal discrimination trials. No sensory discrimination tasks were attempted with more test conditions (e.g., location, quality, intensity, or combinations) than those reported herein.

Discrimination experiments were performed by delivering randomly-ordered stimulation trials in which the subject was required to classify the location, quality, and/or intensity of the evoked percept for each trial. Stimulation conditions varied across trials, including stimulation via different USEA electrodes or combinations of electrodes, and/or use of different stimulation frequencies. The number of electrodes selected for each discrimination study was based on the time remaining in a session and the desire to compare multiple trials of stimulation on each electrode in a blinded, randomized fashion. Formal discrimination trials were preceded by informal practice trials in which the subject experienced each different stimulation condition and formulated category labels for the percept associated with the condition. Once the subject felt comfortable identifying the location, quality, and/or intensity of the different stimulation conditions, formal blind trials commenced in which the subject was required to select one of his predetermined percept categories in response to each stimulation trial. The number of trials per condition was selected to have enough statistical power to detect significant performance on the overall task, not necessarily for each individual condition. The number of trials per condition was selected to be psychometrically realistic so that mental fatigue did not confound the subject’s performance within an experimental session. Cross-session comparisons were not pursued in these initial studies because they can introduce other confounds from differences in percepts evoked by electrode stimulation [54].

A summary of discrimination tasks and the number of USEA electrodes used can be found in Table 2.

**Table 2:** Summary of discrimination tasks

Participant	Discrimination task	Total conditions	Number of trials	Electrode(s)	Percept(s)
S3	Location	5	35	46	D4 tip
				66	D5 tip
				23	Palm
				48	Wrist
				46, 66, 23, 48 (simultaneous)	D4 tip, D5 tip, palm, wrist
S3	Location	4	24	26	Palm
				45	D5 tip
				26, 45 (simultaneous)	Palm, D5 tip, merging sensation
				26, 45 (interleaved)	Palm, D5 tip
S4	Location	8	24	none	none
				16	D5, palm
				28	D5 side,
				36	D4 Tip
				16, 28	D5, palm, D5 side
				16, 36	D5, palm, D4 tip
				28, 36	D5 side, D4 tip
				16, 28, 36	D5, Palm, D5 side, D4 tip
S3	Quality	2	30	39	Tingle
				44	Vibration
S5	Intensity	4	20	none	none
				76 (35 Hz)	Light pressure
				76 (70 Hz)	Medium pressure
				76 (100 Hz)	Heavy pressure
S5	Location-intensity	10	30	none	none
				66 (30 Hz)	D3, light pressure
				66 (70 Hz)	D3, medium pressure
				66 (100 Hz)	D3, heavy pressure
				59 (30 Hz)	Palm, light pressure
				59 (70 Hz)	Palm, medium pressure
				59 (100 Hz)	Palm, heavy pressure
				8 (30 Hz)	D2, light pressure
				8 (70 Hz)	D2, medium pressure
				8 (100 Hz)	D2, heavy pressure
S5	Location-intensity	7	40	none	none
				18 (100 Hz)	D2, 20° flex
				18 (50 Hz)	D2, 50° flex
				18 (150 Hz)	D2, 180° flex
				24 (30 Hz)	D3, 10° flex
				24 (80 Hz)	D3, 90° flex
				24 (150 Hz)	D3, 180° flex

### Location discrimination

For location discrimination trials, electrodes were preferentially down-selected to represent sensations on different gross anatomical hand regions, such as different digits and the palm. When available, electrodes that evoked sensations at the same location but with different qualities were used for quality discrimination trials.

Subject S3 discriminated among five stimulation conditions that evoked sensations at five different hand locations: (1) D4 tip, (2) D5 tip, (3) palm, (4) wrist, (5) combined perception at all four of these locations (Table 2). These percepts were evoked by individual stimulation of four ulnar-nerve-USEA electrodes and simultaneous stimulation of all four of these electrodes, respectively. Stimulation amplitudes for the four electrodes ranged from 14 to 30 μA. A total of 35 discrimination trials were performed by subject S3 for these five conditions.

Subject S3 also discriminated among four different stimulation conditions that evoked sensations at four different hand locations: (1) palm, (2) D5 tip, (3) both palm and D5 tip, (4) palm, D5 tip, and a merging sensation between the palm and D5 tip (Table 2). These percepts were evoked by: (1) individual stimulation of two different ulnar-nerve-USEA electrodes, (2) simultaneous stimulation of the two ulnar-nerve-USEA electrodes (i.e., no time shift between the stimulation pulses on each electrode), and (3) interleaved stimulation of the two ulnar-nerve-USEA electrodes (i.e., a 2.5-ms time shift between the stimulation pulses on each electrode). Stimulation amplitudes for the two electrodes were 23 μA and 20 μA. A total of 25 discrimination trials were performed by subject S3 for these four conditions.

Subject S4 also discriminated among eight different stimulation conditions that evoked sensations at eight different hand locations: (1) no sensations, (2) D5 and palm, (3) D5 side, (4) D4 tip, (5) D5, palm, D5 side, (6) D5, palm, D4 tip, (7) D5 side, D4 tip, (8) D5, palm, D5 side, D4 tip (Table 2). These percepts were evoked by: (1) no stimulation, (2) individual stimulation of each of three ulnar-nerve-USEA electrodes, (3) simultaneous stimulation using each combination of subsets of two of three electrodes, (4) simultaneous stimulation using all three electrodes. Stimulation amplitudes on the three electrodes ranged from 7 to 13 μA depending on the electrode. A total of 24 discrimination trials were performed by subject S4 for these eight conditions.

### Quality discrimination

Subject S3 discriminated between two different stimulation conditions that evoked sensations at the same hand location but with two different qualities: (1) tingle, (2) vibration (Table 2). These percepts were evoked by individual stimulation of two ulnar-nerve-USEA electrodes. Stimulation amplitudes for the two electrodes were 11 μA and 12 μA. A total of 30 discrimination trials were performed by subject S3 for these two conditions.

### Intensity discrimination

Subject S5 discriminated among four different stimulation conditions that evoked sensations at the same hand location but with different self-reported intensities: (1) no pressure, (2) light pressure, (3) medium pressure, (4) heavy pressure (Table 2). These percepts were evoked by stimulating a single median-nerve-USEA electrode at: (1) 0 Hz (no stimulation), (2) 35 Hz, (3) 70 Hz, (4) 100 Hz. The stimulation amplitude used during trials was 25 μA. A total of 20 discrimination trials were performed by subject S5 for these two conditions.

### Combined location and cutaneous intensity discrimination

Subject S5 discriminated among ten different stimulation conditions that evoked sensations at different hand locations with different self-reported intensities: (1) no sensation, (2) D3, light pressure, (3) D3, medium pressure, (4) D3, heavy pressure, (5) palm, light pressure, (6) palm, medium pressure, (7) palm, heavy pressure, (8) D2, light pressure, (9) D2, medium pressure, (10) D2, heavy pressure. These percepts were evoked by individual stimulation of three median-nerve-USEA electrodes with stimulation frequencies of 30 Hz, 70 Hz, or 100 Hz (Table 2). Sham stimulation was also used (i.e., no stimulation), making a total of ten classification categories (three intensities at each of three percept locations, plus sham). Stimulation amplitudes on the three electrodes ranged from 17 to 64 μA depending on the electrode. A total of 30 discrimination trials were performed by subject S5 for these two conditions.

### Combined location and proprioceptive intensity discrimination

Subject S5 also discriminated among seven different stimulation conditions that evoked sensations at different hand locations with different self-reported proprioceptive intensities: (1) no sensation, (2) D2, 20° flex, (3) D2, 50° flex, (4) D2, 180° flex, (5) D3, 10° flex, (6) D3, 90° flex, (7) D3, 180° flex. These percepts were evoked by individual stimulation of two median-nerve-USEA electrodes. Stimulation frequencies for one median-nerve-USEA electrode were 100 Hz, 50 Hz, and 150 Hz. Stimulation frequencies for the other median-nerve-USEA electrode were 30 Hz, 80 Hz, and 150 Hz (Table 2). Sham stimulation was also used (i.e., no stimulation), making a total of seven classification categories (three proprioceptive intensities at each of two percept locations, plus sham). Stimulation amplitudes on the two electrodes were from 17 to 40 μA. A total of 40 discrimination trials were performed by subject S5 for these seven conditions.

### Data analysis

Discrimination trial results reported here were not pooled across sessions or subjects. Data analysis for discrimination trials was performed using the binomial test, where the probability of guessing the correct classification on a given trial was determined as the inverse of the number of predetermined classification categories. Post-hoc analyses included removal of the sham “no stimulation” condition in order to isolate the discriminability of percepts at higher stimulus intensities. Hypothesis testing was performed with a critical value of α = 0.05. A Bonferroni adjustment was made to the critical value for any additional post-hoc tests by dividing the critical value by the number of post-hoc tests performed.

## Results

All subjects were able to discriminate percepts by location, quality, and intensity with performance significantly greater than chance. Additionally, subject S5 performed combined location/quality/intensity discrimination trials, including trial sets with cutaneous percepts and trial sets with proprioceptive percepts. Discrimination among percepts of different locations, qualities, and intensities will be important for future use of sensory feedback from multiple prosthesis-coupled sensors during closed-loop prosthesis control.

### Location discrimination

**Fig. 2:**
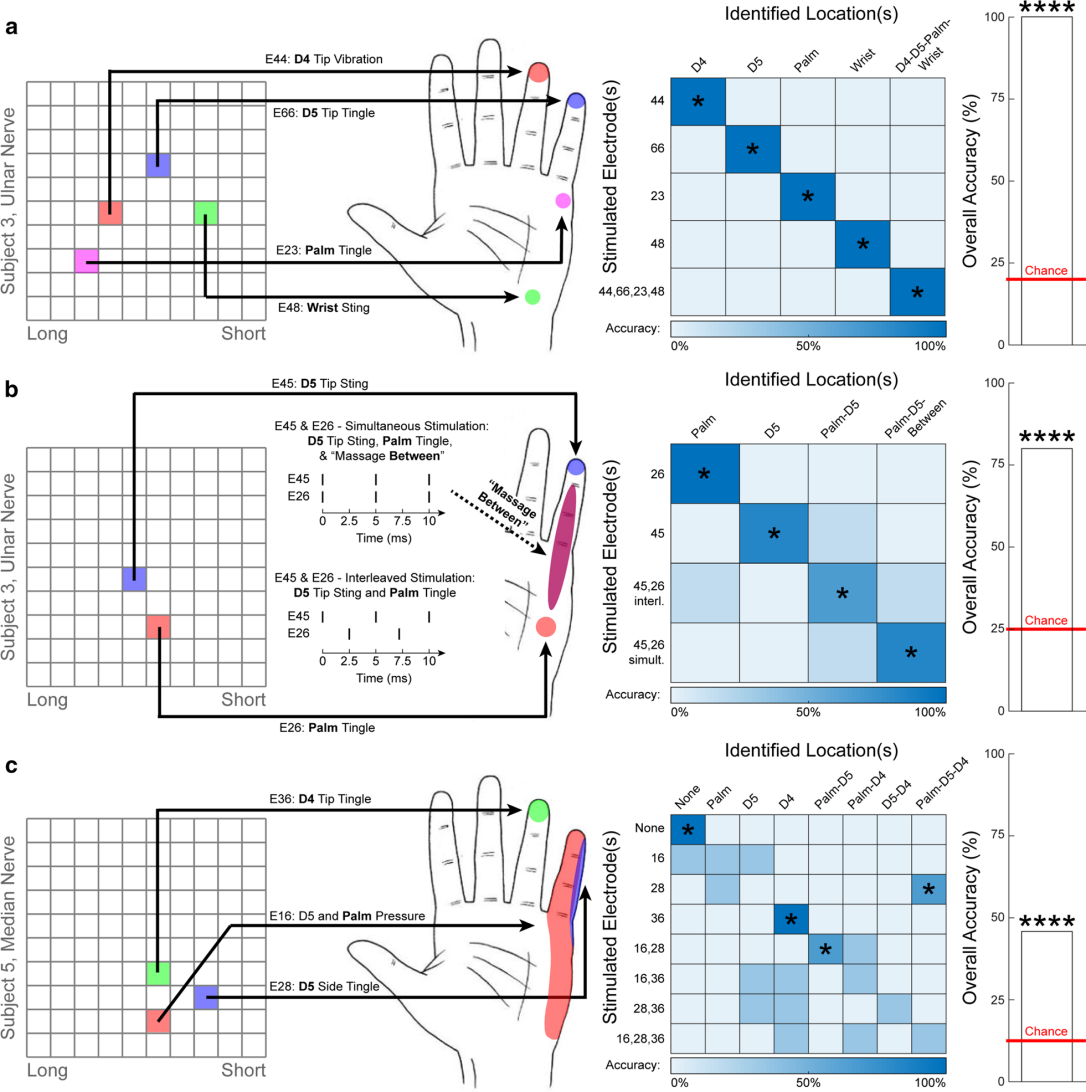
Location discrimination trials. Each sub-figure depicts the location of electrodes which evoked different hand sensations, as well as a confusion matrix showing the discrimination performance, and the overall accuracy relative to chance. Full descriptions of the percepts are presented next to the image of the hand and are abbreviated to the bold word in the confusion matrix. Confusion matrices are square, such that the correct answers lie along the diagonal. Asterisks over the bar plot indicates statistical significance with regards to the aggregate data (primary outcome measure). Asterisks overlaid on the confusion matrix indicate statistical significance with regards to an individual condition (secondard outcome measure with limited statistical power). **a** Subject S3 successfully discriminated among percepts evoked via individual stimulation of four different ulnar-nerve-USEA electrodes, as well as simultaneous stimulation of all four electrodes (four categories shown, the fifth category was concurrent perception at all four locations; 35/35 successful trials, *p* < 0.0001, binomial test). **b** Subject S3 also discriminated successfully between individual stimulation of two ulnar-nerve-USEA electrodes (separate solid arrows), as well as simultaneous interleaved stimulation (same as solid arrows) and interleaved stimulation (solid arrows plus dotted arrow) of the same two ulnar-nerve-USEA electrodes. Interleaved stimulation (2.5-ms time shift difference, 200 Hz) reproduced the original percepts simultaneously with no merging sensation, whereas simultaneous stimulation (no time shift difference, 200 Hz) produced both of these percepts accompanied by an merging sensation between them (20/24 successful trials, *p* < 0.0001, binomial tests). Timing of individual stimulation pulses is shown as a raster plot to the left of the hand. **c** Subject S4 discriminated among eight different stimulation configurations: individual stimulation of each of three ulnar-nerve-USEA electrodes, simultaneous combined stimulation using different subsets of two of these three electrodes, simultaneous combined stimulation using all three electrodes, and no stimulation (11/24 correct trials, p < 0.0001, binomial test). Importantly, these trials with S4 also included a condition of “no stimulation,” which was identified with 100% accuracy, indicating that percepts were indeed evoked by USEA stimulation (in contrast to pseudesthesia). These three experiments also demonstrate the selectivity of USEA-electrode stimulation, with unique percepts being generated by electrodes as close as 800 μm. * = *p* < 0.05, ** = *p* < 0.01, *** = *p* < 0.001, **** = *p* < 0.0001; overall binomial test

Subject S3 discriminated among five stimulation conditions that evoked sensation at five different hand locations. The subject discriminated among these stimulation conditions by classifying the percept evoked into one of the five predetermined classification categories in 35/35 successful trials (20% chance per trial, *p* < 0.0001, binomial test; Fig. 2a). Importantly, the four electrodes selected for these stimulation trials had tip positions as close as ~ 899 μm within the nerve, yet they each consistently evoked unique sensory percepts, suggesting selectivity in axon activation. Additionally, the combined stimulation of all four electrodes did not result in emergent sensory percepts (a percept with different quality or location from the four individual percepts), suggesting that current summation during simultaneous stimulation was limited.

To better study current summation during simultaneous stimulation of multiple electrodes in subject S3, we selected two ulnar-nerve-USEA electrodes with tips placed less than ~ 899 μm apart within the nerve (~ 805 μm cross-sectional projection separation assuming USEAs were implanted squarely perpendicular to the nerve) and delivered four stimulation conditions: (1 & 2) individual stimulation of two different ulnar-nerve-USEA electrodes, (3) simultaneous stimulation of the two ulnar-nerve-USEA electrodes (i.e., no time shift between the stimulation pulses on each electrode), and (4) interleaved stimulation of the two ulnar-nerve-USEA electrodes (i.e., a 2.5-ms time shift between the stimulation pulses on each electrode) (Fig. 2b). The individual stimulation via two different electrodes consistently produced sensations of D5-tip sting and lateral-palm tingle, respectively, whereas interleaved stimulation of these electrodes (2.5-ms time shift difference) consistently reproduced both of these percepts concurrently with no emergent sensations, and simultaneous stimulation (no time shift difference) consistently produced both of these percepts concurrently accompanied by an emergent ‘massage’ feeling bridging between them. The participant successfully identified among these four sensations in 20/24 trials (25% chance per trial, *p* < 0.0001, binomial test; Fig. 2b).

Subject S4 also performed location-discrimination trials, including discrimination among eight different cutaneous stimulation configurations (Fig. 2c). The participant successfully identified between these sensations in 11/24 trials (12.5% chance per trial, *p* < 0.0001, binomial test, Fig. 2c). Importantly, these trials also included a condition of “no stimulation,” to validate that percepts were indeed evoked by USEA stimulation in contrast to pseudethesia. Subject S4 successfully identified when stimulation was delivered compared with when no stimulation was delivered in 24/24 trials (50% chance per trial, *p* < 0.0001, binomial test). Furthermore, even when the “no stimulation” condition was removed in a post-hoc analysis, the participant still successfully discriminated among the evoked percepts in 8/21 trials (14.2% chance per trial, *p* < 0.01, binomial test).

Subject S4 also performed location-discrimination trials with both single-channel and multichannel stimulation. The participant successfully identified the location when single-channel stimulation was used in 4/9 trials (12.5% chance per trial, *p* < 0.05, binomial test), and when multichannel stimulation was used in 5/12 trials (12.5% chance per trial, *p* < 0.05, binomial test). We found no significant different between these two stimulation conditions (4/9 versus 5/12, p = 0.89, chi-squared test), although statistical power is lacking to rule out false negatives.

### Quality discrimination

**Fig. 3:**
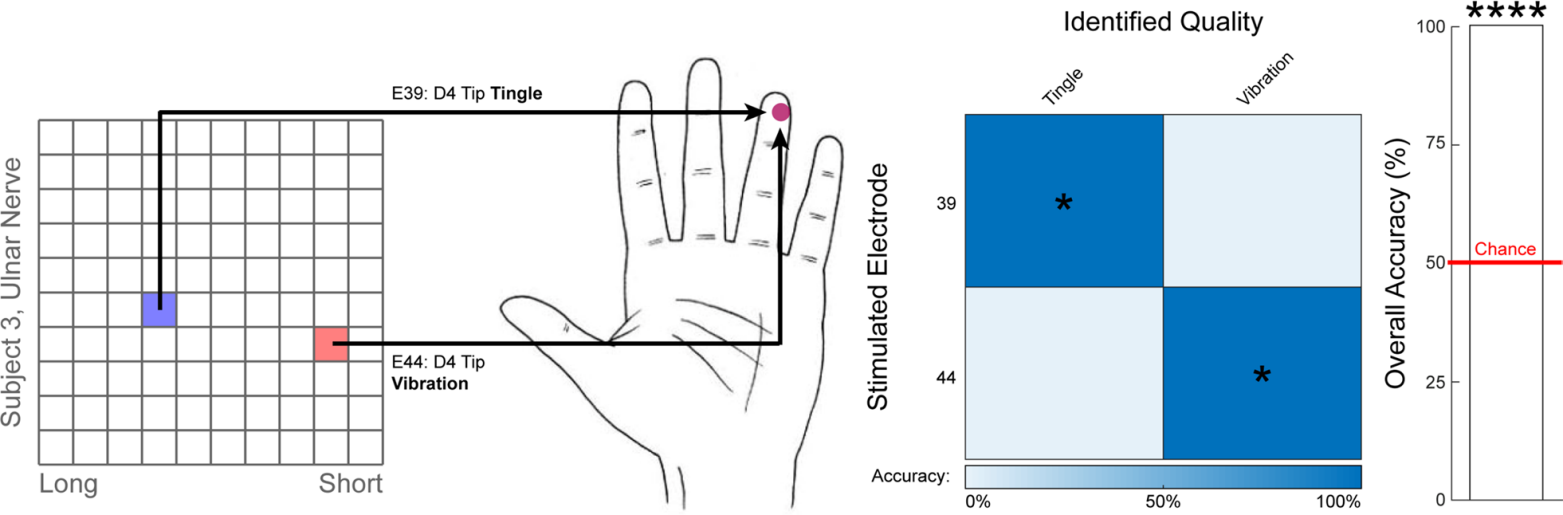
Quality discrimination trials. Figure depicts the location of electrodes which evoked different hand sensations, as well as a confusion matrix showing the discrimination performance, and the overall accuracy relative to chance. Full descriptions of the percepts are presented next to the image of the hand and are abbreviated to the bold word in the confusion matrix. Confusion matrices are square, such that the correct answers lie along the diagonal. Asterisks over the bar plot indicates statistical significance with regards to the aggregate data (primary outcome measure). Asterisks overlaid on the confusion matrix indicate statistical significance with regards to an individual condition (secondard outcome measure with limited statistical power). Subject S3 successfully discriminated between stimulation of two different USEA electrodes that evoked sensation at the same location, but with different qualities (vibration versus tingle). Regarding the locations of the two percepts, the subject said they were “Right on, exact same space.” He also indicated that these sensory percepts were the same intensity level. The subject successfully performed the classification in 30/30 trials (p < 0.0001, binomial test). * = *p* < 0.05, ** = *p* < 0.01, *** = *p* < 0.001, **** = *p* < 0.0001; binomial test

Subject S3 successfully discriminated between two evoked percepts with the same perceived somatotopic location, but with two distinct qualities, produced via stimulation of two different ulnar-nerve-USEA electrodes (Fig. 3). Prior to formal discrimination trials, the subject identified the percepts evoked by these two different electrodes as having identical intensities and locations near the D5 tip (“Right on, exact same space”), but differing qualities of vibration and tingle, respectively. In subsequent formal trials, the subject consistently discriminated between the percepts evoked by the two electrodes in 30/30 trials (50% chance per trial, *p* < 0.0001, binomial test).

### Intensity discrimination

**Fig. 4:**
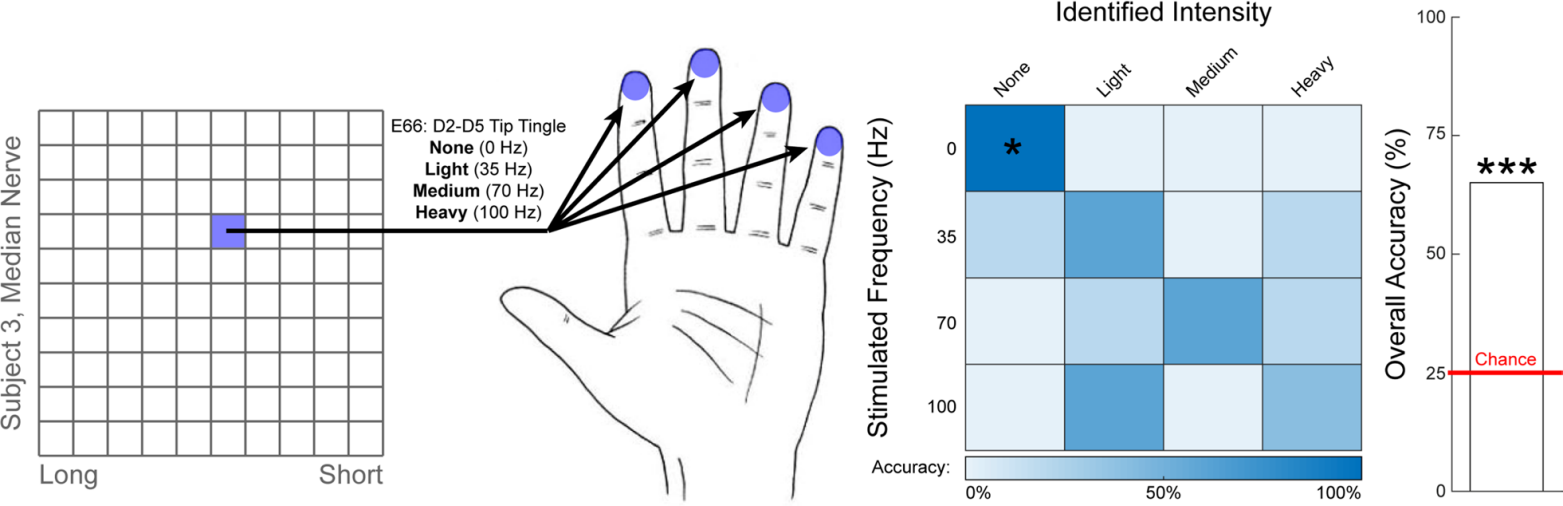
Intensity discrimination trials. Figure depicts the location of electrodes which evoked different hand sensations, as well as a confusion matrix showing the discrimination performance, and the overall accuracy relative to chance. Full descriptions of the percepts are presented next to the image of the hand and are abbreviated to the bold word in the confusion matrix. Confusion matrices are square, such that the correct answers lie along the diagonal. Asterisks over the bar plot indicates statistical significance with regards to the aggregate data (primary outcome measure). Asterisks overlaid on the confusion matrix indicate statistical significance with regards to an individual condition (secondard outcome measure with limited statistical power; *p* = 0.08 for 35 Hz and 70 Hz conditions). Subject S5 discriminated between four percept intensities, evoked by stimulation of a single median-nerve-USEA electrode at three different frequencies (35 Hz, 70 Hz, 100 Hz) or sham (no stimulation). The evoked sensory percept was described as ‘tingle’ on all four fingertips. The subject successfully classified these different intensities in 13/20 trials (p < 0.0005, binomial test). * = *p* < 0.05, ** = *p* < 0.01, *** = *p* < 0.001, **** = *p* < 0.0001; binomial test

Subject S5 successfully discriminated among four different cutaneous-percept intensities, encoded via stimulation with different frequencies on a single USEA electrode (Fig. 4). During informal practice trials, the subject designated four intensity levels as “high,” “medium,” “light,” or “nothing,” corresponding to stimulation at 100 Hz, 70 Hz, 35 Hz or no stimulation, respectively. During subsequent formal trials, the subject correctly classified these percept intensities in 13/20 trials (25% chance per trial, *p* < 0.0005, binomial test, Fig. 4). With the “no stimulation” condition removed in a post-hoc analysis, the participant’s performance was no longer statistically significant, although a trend toward statistical significance was observed (8/15 trials correct; 33% chance per trial, *p* = 0.08, binomial test).

### Combined location and cutaneous intensity discrimination

**Fig. 5:**
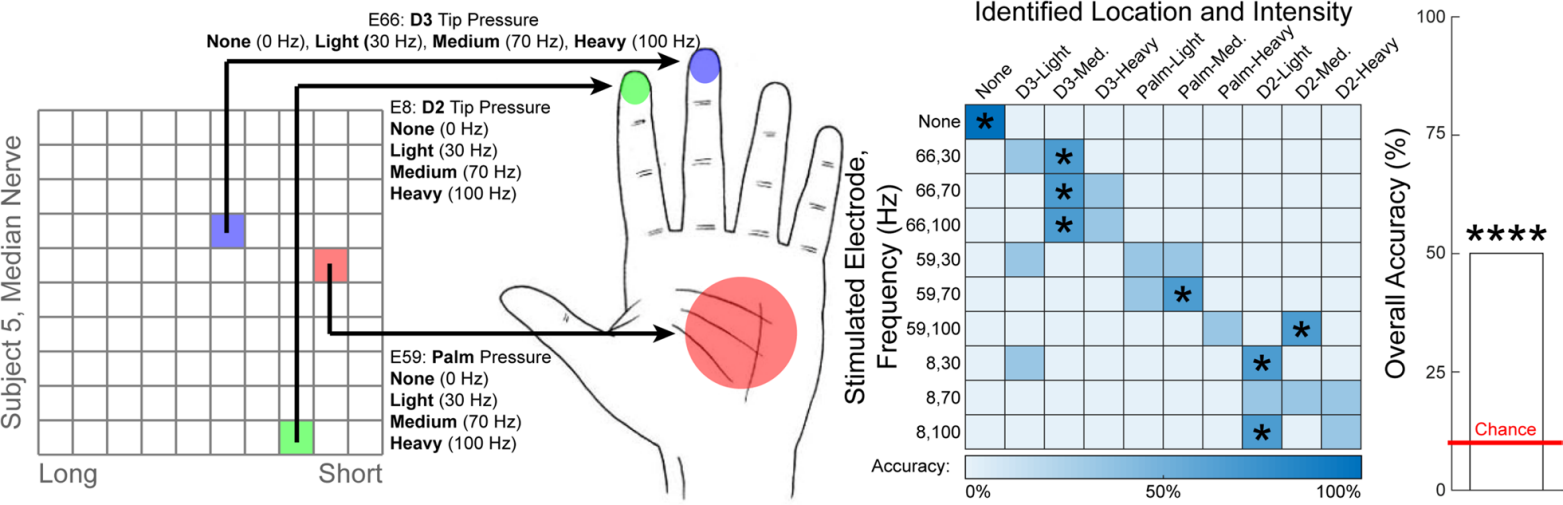
Combined cutaneous location and intensity discrimination. Figure depicts the location of electrodes which evoked different hand sensations, as well as a confusion matrix showing the discrimination performance, and the overall accuracy relative to chance. Full descriptions of the percepts are presented next to the image of the hand and are abbreviated to the bold word in the confusion matrix. Confusion matrices are square, such that the correct answers lie along the diagonal. Asterisks over the bar plot indicates statistical significance with regards to the aggregate data (primary outcome measure). Asterisks overlaid on the confusion matrix indicate statistical significance with regards to an individual condition (secondard outcome measure with limited statistical power). Subject S5 discriminated among combinations of different cutaneous percept locations and intensities. Three median-nerve-USEA electrodes evoked cutaneous “pressure” percepts on D2, D3, and the palm, respectively. Three frequencies (35 Hz, 70 Hz, and 100 Hz) were used to encode three different intensities via each electrode. Sham trials were also included (no stimulation) for a total of ten classification categories. The subject correctly classified the combination in 15/30 trials (p < 0.0001, binomial test). In post-hoc analysis, we found that most of the subject’s success was attributed to accurate location discrimination (26/30 correct trials, p < 0.0001, binomial test for location classification independent of intensity classification, using a corrected critical value of α = 0.005). * = *p* < 0.05, ** = *p* < 0.01, *** = *p* < 0.001, **** = *p* < 0.0001; binomial test

Subject S5 performed combined location- and intensity-discrimination trials for cutaneous percepts, similar to what may be used as part of a multi-sensor closed-loop prosthesis (Fig. 5). Subject S5 successfully discriminated among ten different stimulation conditions in 15/30 trials (10% chance per trial, *p* < 0.0001, binomial test, Fig. 5). Furthermore, even when the “no stimulation” condition was removed in a post-hoc analysis, the participant still successfully discriminated among the evoked percepts in 12/27 trials (11.1% chance per trial, *p* < 0.0001, binomial test). In post-hoc analyses, we found that most of the subject’s success was attributed to accurate location discrimination; location was identified correctly in 26/30 trials (25% chance per trial, *p* < 0.0001, binomial test for location classification independent of intensity classification, using a corrected critical value of α = 0.005). In contrast, intensity discrimination was successful but seemed more challenging; intensity was identified correctly in 17/30 trials (25% chance per trial, *p* < 0.0005, binomial test for intensity classification independent of location classification, using a corrected critical value of α = 0.005).

### Combined location and proprioceptive intensity discrimination

**Fig. 6:**
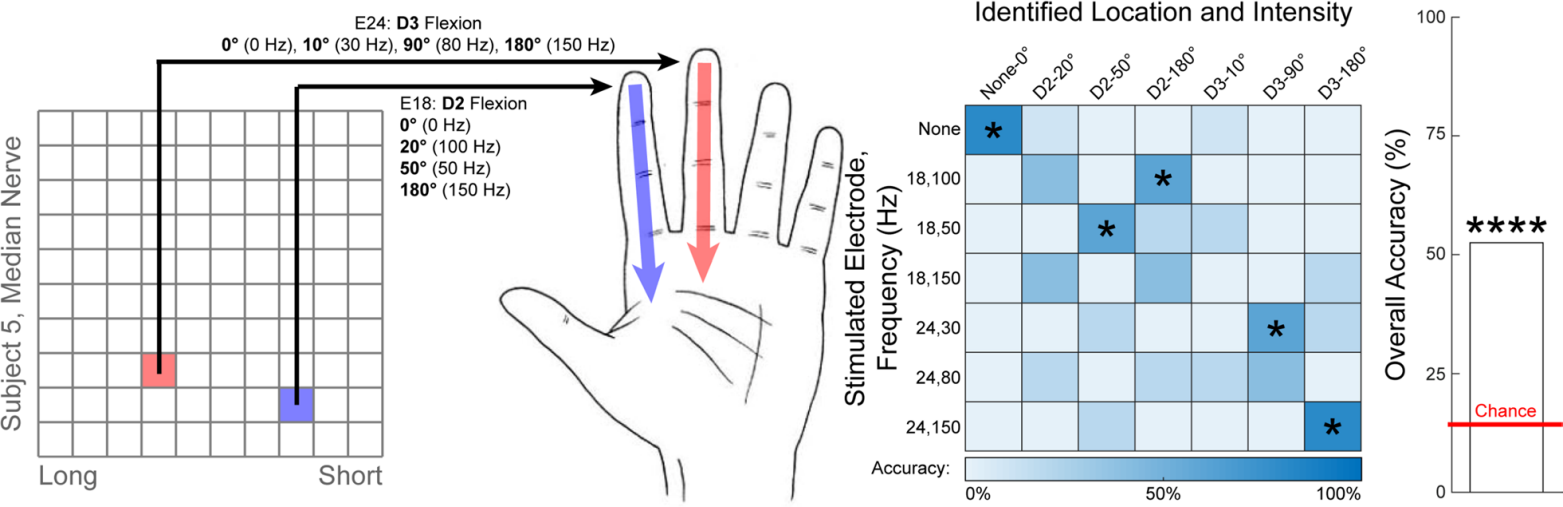
Combined proprioceptive location and quality discrimination. Figure depicts the location of electrodes which evoked different hand sensations, as well as a confusion matrix showing the discrimination performance, and the overall accuracy relative to chance. Full descriptions of the percepts are presented next to the image of the hand and are abbreviated to the bold word in the confusion matrix. Confusion matrices are square, such that the correct answers lie along the diagonal. Asterisks over the bar plot indicates statistical significance with regards to the aggregate data (primary outcome measure). Asterisks overlaid on the confusion matrix indicate statistical significance with regards to an individual condition (secondard outcome measure with limited statistical power). Subject S5 discriminated between combinations of different proprioceptive percept locations and intensities. Two median-nerve-USEA electrodes evoked perception of proprioceptive flexion of D2 or D3. Three frequencies were used on each electrode to encode three different joint positions. Sham trials were included (no stimulation) representing a fully-open rest position for a total of seven classification categories. The subject correctly classified 21/40 trials (p < 0.0001, binomial test). * = *p* < 0.05, ** = *p* < 0.01, *** = *p* < 0.001, **** = *p* < 0.0001; binomial test

Subject S5 performed combined location- and intensity-discrimination trials for proprioceptive percepts, similar to what may be used as part of a multi-sensor closed-loop prosthesis (Fig. 6). Subject S5 successfully discriminated among seven different stimulation conditions in 21/40 trials (14.3% chance per trial, *p* < 0.0001, binomial test). Furthermore, even when the “no stimulation” condition was removed in a post-hoc analysis, the participant still successfully discriminated among the evoked percepts in 13/30 trials (16.6% chance per trial, p < 0.001, binomial test). The subject correctly identified which phantom digit moved in 32/40 trials (50% chance per trial, *p* < 0.0001, post-hoc binomial test for digit classification independent of joint-position classification, using a corrected critical value of α = 0.005). The subject identified the correct joint position in 22/40 trials (33% chance per trial, *p* < 0.005, binomial test for joint-position classification independent of joint classification, using a corrected critical value of α = 0.005).

### Qualitative descriptions of sensory percepts

The subjects generally appreciated the cutaneous and proprioceptive sensations evoked by USEA stimulation, although a small percentage of USEA electrodes can evoke painful or uncomfortable sensations associated with nociceptive sensory fibers [54]. After his first stimulation session, subject S3 stated, “My hand is starting to stimulate like it’s starting to wake up or something. It really feels good. […] It’s good to know that there’s something still there.” We did not formally quantify the naturalism of the evoked sensory percepts. However, informal remarks and comparisons to every-day sensory experiences suggest that most percepts were perceived as somewhat natural. For example, in response to the proprioceptive percept of D3 flexion delivered during proprioception discrimination trials, subject S5 stated that the sensation felt “exactly like movement of the middle finger.” Similarly, when asked to describe one of the sensory percepts evoked during cutaneous location-intensity discrimination trials, subject S5 stated, “It feels like touch. It feels like if I touched that door.”

## Discussion

We have demonstrated that USEA stimulation can be used to encode sensory percepts with different locations, qualities, and intensities that can be reliably discriminated with a performance greater than chance alone. Further, discrimination was possible for both cutaneous and proprioceptive percepts. Encoding of cutaneous sensory percepts with different locations and qualities was achieved by stimulation of different USEA electrodes or combinations of electrodes, presumably resulting in activation of different axons or subsets of axons within the nerve. Encoding of sensory percepts with different intensities was achieved by modulation of the stimulation frequency, presumably resulting in an increased firing rate in activated axons [24]. We have also demonstrated that subjects can discriminate among multiple location-intensity combined percepts such as would be desired during closed-loop prosthesis control.

Importantly, we demonstrated the ability to selectively evoke percepts with the same location but different qualities. It has been shown that modulating stimulation frequency increases percept intensity but not location or quality. Increasing amplitude also increases intensity, but by recruiting additional sensory fibers, that may be associated with different qualities. We hypothesize that the percepts with overlapping projected fields but different qualities were evoked by activating two different sensory afferent subtypes. This result suggests that subjects may be able to discriminate among activation of different afferent subtypes that have overlapping projected fields. The ability to selectively evoke percepts with overlapping projected fields and different qualities may allow for fiber-level biomimetic sensory feedback [55].

Additionally, we show that subjects can discriminate between simultaneous and interleaved multi-electrode stimulation. The subject was able to reliably feel an emergent massage feeling during simultaneous multi-electrode stimulation but not with interleaved multi-electrode stimulation. One plausible explanation for the emergent massage feeling during simultaneous stimulation is that multiple additional axons may have been activated due to spatiotemporal current summation from the two electrodes [47]. Future use of simultaneous and interleaved multielectrode stimulation may allow for improvements in the number and nature of restored percepts. This result also provides an important proof-of-concept for a method of interleaving stimulation via different USEA electrodes when current-summation effects are not desired, for example, during closed-loop prosthesis control with simultaneous USEA-evoked sensory feedback from multiple prosthesis sensors. Although we have shown that USEA electrodes as close as 800 μm within the nerve cross-section can evoke distinct sensory percepts, simultaneous stimulation via these electrodes often results in current summation and potentially undesired activation of additional axons that evoke additional sensation. Use of interleaved stimulation allows for simultaneous generation of the individual sensory percepts without current-summation effects. During closed-loop prosthesis control, interaction with the external environment may result in simultaneous activation of multiple prosthesis sensors, potentially generating simultaneous stimulation via multiple USEA electrodes. Algorithms may be developed and incorporated to interleave stimulation on different USEA electrodes to prevent current-summation effects. One tradeoff of interleaving stimulation is that a more frequent occurrence of stimulation artifact will likely be produced in USEA electrode recordings, possibly interrupting the ability to decode neural recordings for prosthesis control. In this case, it may be desirable to develop stimulation artifact blanking approaches or to implant separate recording electrodes in a distant location where stimulation artifact will be minimized (e.g., the residual limb muscles or a distant nerve location).

It is uncertain in some cases whether proprioceptive percepts were elicited by activating proprioceptive fibers directly or by generating secondary proprioceptive signaling after activating motor fibers. In the majority of cases, we did not observe visible muscle twitching in the residual limb during USEA stimulation; however, there is a possibility that single motor fibers were activated without producing a visible muscle twitch. However, proprioceptive percepts associated with movements of missing hand muscles (e.g., intrinsic hand muscles) do not suffer from this potential confound and hence are likely due to direct activation of proprioceptive fibers. Furthermore, subject S5 described both monotonic and nonmonotonic frequency-intensity encoding for D3 and D2 joint positions, respectively. These activations patterns are consistent with those that have been previously reported for muscle spindle fibers [56, 57].

Sensory feedback from the hand has been shown to be important for identifying when contact events between the hand and the environment occur and for identifying object properties such as curvature, texture, and weight. These complex properties are interpreted using sensory integration across various proprioceptive and cutaneous channels with many receptive fields. Cutaneous information, encoded via multiple different receptors (e.g., slowly-adapting I, slowly-adapting II, rapidly-adapting I, and rapidly-adapting II), provides information regarding contact locations, object texture, object slippage, and gross shape [41, 43, 58–61]. Proprioceptive channels provide information regarding hand conformation and position, which, in conjunction with cutaneous information, provides information regarding object shape, weight, and counterforce [62]. Many of these object properties are challenging to deduce using visual feedback alone, particularly when feedback is needed rapidly during motor tasks [63] or when handling opaque objects. The goal of discrimination among a variety of sensory channels is ultimately to provide the brain with sufficient information to deduce useful information regarding interactions with the external environment.

Our gross encoding of three stimulus locations, each with three different intensities, may be sufficient to assist subjects in identifying gross object properties such as size and compliance [4]. However, more complex properties such as curvature and skin indentation direction and force gradations will likely require encoding via sensory percepts of different submodalities (e.g., rapidly-adapting I and slowly-adapting I) that have nearby projected fields [64]. Restored sensation via multiple axons with adjacent projected fields may be critical for naturalistic sensorimotor hand control because real-time neural encoding of object properties likely involves cortical comparison of spike timings from neurons with adjacent receptive fields [65]. We anticipate that functional prosthesis control will improve with increasing number and variety of discriminable sensory feedback channels. Importantly, the data reported in this paper represents the most complex sensory discrimination tasks attempted with subjects S4 and S5; future studies should be designed to attempt more sophisticated discrimination tasks.

The ultimate goal of restored prosthesis sensation is not just to provide subjects with a useful tool, but also to provide subjects with a prosthesis that is perceived by subjects as a replacement hand. Although the results of this report do not begin to approximate the sophistication of an intact hand (hundreds of discriminable cutaneous locations, and ~ 50 discriminable force levels), this work constitutes an important step in demonstrating the capabilities of USEAs to restore a variety of discriminable sensory percepts, which may ultimately help guide the development of sensorized bionic arms. Specifically, we have demonstrated that different USEA-evoked percepts are discriminable from each other, including up to three gross-level hand regions such as different digits and the palm, each with three different intensities. These results are comparable to a recent study in which transfemoral amputees were able to discriminate among a combination of two or three cutaneous percepts and two or three emulated proprioceptive percepts (stimulation of efferent nerves to indirectly activate proprioceptive fibers) evoked by intraneural stimulation [66]. Likewise, the results presented here are similar to those reported by another recent study in which transradial amputees discriminated among seven different hand postures evoked by epineural stimulation [36].

The intensity discrimination results presented here show the participant was capable of discriminating among 0 Hz, 35 Hz, 70 Hz and 100 Hz at a level greater than chance alone. However, when the 0-Hz condition was removed in a post-hoc analysis, the participant’s ability to discriminate among the three remaining intensity levels (low, medium and high) was almost but not quite statistically significant (p = 0.08). The ability to discriminate a 30-Hz difference at a 100-Hz reference with at least 75% accuracy requires a Weber fraction of 0.3 for changes in intraneural pulse frequency. This Weber fraction is consistent with those reported previously for intraneural stimulation via USEAs [46], as well as for those reported for epineural stimulation [16, 24]. Thus, we speculate that with additional data the trend observed (p = 0.08) would reveal significance, even with the “no stimulation” condition removed. In real-world activities of daily living, the ability to determine whether a stimulus is absent or present is among the most important discriminative capabilities. Removal of the “no stimulation” condition here is an artificial experimental constraint intended to isolate the discriminability of percepts at higher stimulus intensities.

It is also important to note that the discrimination tasks presented here were designed to be psychometrically ergonomic and to be analyzed at the aggregate level. As such, we did not have enough trials at each individual condition to eliminate the possibility of false negatives when looking for statistically significant performance on the individual conditions. Future studies should increase the number of trials at each condition by performing the task across multiple days after ensuring cross-session stability of the evoked percepts.

Although the results presented here do not recreate the performance of intact human hands, this level of discriminability has been shown to result in substantial functional improvements in fragile object manipulation and haptic perception [1, 4], and performance may improve with long-term use [25, 67]. Prior work has highlighted hundreds of sensory percepts from USEAs [13, 14, 54], but these studies did not test for false positives using sham conditions. In contrast, here we show that subjects can reliably discriminate sensory percepts from one another, and importantly, from sham conditions. However, infrequently but occasionally subjects were not able to identify the correct answer at all (e.g., Fig. 2c, 0% accuracy for electrode 28). Given that the subject consistently described the percept by a description different from what had been originally proposed, we speculate that the percept may have changed from when it was given its “correct” label during informal practice trials to when the participant was attempting to identify the “correct” label during formal discrimination trials.

Ongoing work should focus on discrimination among successively closer projected fields to identify minimum discriminable distances. Additionally, interleaved, multielectrode stimulation strategies may produce surround inhibition effects that could improve percept discrimination. Although USEAs offer the highest channel count of any peripheral nerve interface, 100 channels likely will not provide the incredibly fine level of resolution that would be required to completely restore sensory hand function. Development of a neural interface that may provide such resolution remains as a substantial challenge to the field.

One limitation of this report is the lack of control of the intensity and/or quality of sensory percepts during location-only discrimination trials. Specifically, subjects described the different sensory percepts by indicating both their location and their quality, raising the possibility that the discrimination may not have been performed based on location alone. Future studies regarding location discrimination should attempt to control for the quality and intensity of the percepts.

We have also demonstrated in this report that selective activation of distinct axons or subsets of axons is possible using USEA electrodes as close as ~ 800 μm within the nerve. Stimulation amplitudes were between 7 and 64 μA for the trials reported here, which apparently allowed for focal activation of a subset of axons within the local area of an electrode tip that was distinct from the subset of axons activated by electrodes ~ 800 μm away. Future testing should be performed using closer electrodes, such as neighboring electrodes that are ~ 400 μm apart, to see if selectivity is achievable. Additionally, we anticipate that selectivity will decrease primarily as a function of cross-sectional projection distance, suggesting that electrodes that are directly distal/proximal to each other are less likely to evoke selective sensory percepts due to the possibility that the same axon(s) will pass near each electrode tip as they travel longitudinally along the course of the nerve. More data from electrodes with a variety of different cross-sectional projection distances is needed to perform such an assessment. Future USEA designs may use a steeper slant to allow for improved selectivity along distal–proximal rows.

Informally, we observed adaptation of some sensory percepts during intensity discrimination trials in subject S5. We did not explicitly study the adaptation phenomenon extensively here. However, prior research has documented this phenomenon for neural stimulation [26], and adaptation of responses evoked by natural sensory stimuli is ubiquitous [68, 69]. Informally, we found that in some cases there seemed to be less nominal adaptation if the stimulation was delivered at an amplitude at least 150% of threshold, and if we allowed for ~ 30 s of rest between each trial. Despite this, we informally observed that the subject’s performance discriminating among intensities declined slightly as trials continued, and the subject tended to underestimate the percept intensity in later trials compared with earlier trials in a session. The adaptation of USEA-evoked sensory percepts should be studied and understood explicitly in future studies, particularly for longer-duration sensory prosthesis use. Intact subjects exhibit adaptation in response to tactile stimulation of the skin [70–74]. Past studies indicate that the rate of nominal habituation varies as a function of stimulus frequency and inversely as a function of stimulus strength for neural interface stimulation [23, 26], suggesting that algorithms that use either frequency or charge per pulse to encode stimulus intensity may have different consequences across time, even though the two parameters may be functionally interchangeable for a given time point [31]. We hypothesize that use of suprathreshold stimulation intensities, or addition of interpulse variability into stimulation trains (in contrast to constant-frequency stimulation), to produce more biomimetic stimulation patterns [4, 21], may help reduce the effects of adaptation.

Although the sensory percepts restored via USEA stimulation are generally stable within a 2–3 h session, the projected field location, quality, and intensity associated with each electrode often varies across sessions [13, 54]. Due to this limitation, we did not attempt to repeat identical discrimination tasks in different sessions. This instability may be due to a number of factors, including micromechanical shifts of the USEA relative to nerve fibers, the developing foreign body response to implanted USEAs, or degradation or failure of USEA electrodes and/or wire bundles [75]. A complete characterization of how USEA-evoked percepts and discriminability change throughout long-term implant durations was outside the scope of this study, and warrants further investigation. Furthermore, the subjects involved in this study had USEAs implanted for a total of 4, 5, and 13 weeks. Recent reports have now documented the long-term performance of USEAs implanted for over 70 weeks [54].

Ultimately, we foresee development of a closed-loop prosthesis system with multiple discriminable sensory percepts coupled to sensors that span a physical prosthetic hand for use in activities of daily living. We anticipate that discriminable sensory feedback via a prosthesis will enhance motor control, particularly in scenarios where visual feedback is limited or undesired. Also, we anticipate that discriminable, multi-sensor feedback with variable intensity and tunable quality will further enhance the level of embodiment of a prosthetic limb, helping amputees to feel as though their prosthesis is a replacement hand, in addition to being a useful tool. Sensory feedback during closed-loop control, and any associated limb embodiment, may also alleviate phantom pain and many of the psychological difficulties associated with losing a hand [3, 20, 23, 25].

## Conclusion

We have shown that human amputees implanted with USEAs in their residual peripheral arm nerves can discriminate among a variety of restored hand sensations in blind trials, including: (a) percepts with different hand locations, (b) percepts with different qualities, and (c) percepts with different intensities. Additionally, we have demonstrated that one subject was able to discriminate among cutaneous or proprioceptive percepts with different combinations of location and intensity, such as may occur during functional prosthesis use with multiple graded sensors for feedback. Furthermore, we have presented a multielectrode stimulation strategy using interleaved stimulation, which may be useful for evoking multiple sensory percepts concurrently without the effects of current summation during closed-loop prosthesis control. The subjects enjoyed most of the sensory percepts and appreciated feeling controlled sensation from their amputated hand. Future work should include investigation of discriminability using multielectrode biomimetic stimulation patterns, as well exploration of the limit of discriminability resolution with USEAs. We hypothesize that increasing the number of sensory percepts that can be discriminated by location, quality, and intensity during closed-loop prosthesis control will further increase embodiment and motor performance for prosthesis users.

## Data Availability

All data needed to evaluate the conclusions are available in the paper or the supplementary materials. Data and materials requests should be sent to G.A.C. (greg.clark@utah.edu). Requestors may need to be approved by the human-subjects research committees (e.g., local institutional review board and Department of the Navy Human Resources Protection Program) to comply with Health Insurance Portability and Accountability Act requirements.

## Abbreviations

USEA: Utah slanted electrode array
JND: Just-noticeable difference
